# Prognostic impact of the preoperative hemoglobin A1c levels in patients with gastric cancer surgery depends on postoperative complications

**DOI:** 10.1007/s00595-020-02103-6

**Published:** 2020-08-08

**Authors:** Jun Shibamoto, Katsutoshi Shoda, Takeshi Kubota, Toshiyuki Kosuga, Hidemasa Kubo, Takuma Ohashi, Hiroki Shimizu, Tomohiro Arita, Yusuke Yamamoto, Hirotaka Konishi, Ryo Morimura, Atsushi Shiozaki, Yoshiaki Kuriu, Hisashi Ikoma, Hitoshi Fujiwara, Kazuma Okamoto, Eigo Otsuji

**Affiliations:** Division of Digestive Surgery, Department of Surgery, Kyoto Prefectural University of Medicine, 465 Kawaramachi Hirokoji Kajii-cho, Kamigyo-ku, Kyoto, 602-8566 Japan

**Keywords:** Gastric cancer, Hemoglobin A1c, Prognosis, Postoperative complications

## Abstract

**Purpose:**

The long-term prognostic impact of the hemoglobin A1c levels has not yet been evaluated in patients with gastric cancer. The present study investigated the clinical significance of the hemoglobin A1c levels in patients with gastric cancer.

**Methods:**

We enrolled 294 patients with stage II, III, or IV gastric cancer who underwent gastrectomy. The patients were divided into high preoperative hemoglobin A1c (> 6.0%) and low preoperative hemoglobin A1c (≤ 6.0%) groups.

**Results:**

In patients with stage III gastric cancer with severe postoperative complications, the high preoperative hemoglobin A1c group had a significantly worse prognosis than the low preoperative hemoglobin A1c group (*p* = 0.0409). In patients without severe postoperative complications, the high preoperative hemoglobin A1c group had a significantly favorable prognosis compared with the low preoperative hemoglobin A1c group (*p* = 0.0348).

**Conclusion:**

The prognosis of patients with stage III gastric cancer having high preoperative hemoglobin A1c levels greatly depended on the presence or absence of postoperative complications. To avoid postoperative complications, optimal perioperative management and personalized treatments are critical, particularly for these patients.

## Introduction

Gastric cancer (GC) is the fifth-most frequently occurring malignancy, and it is the third-leading cause of cancer-related deaths worldwide [1]. Despite advances in surgical techniques, postoperative management, and treatment strategies, the long-term prognosis has not significantly improved, which remains a major challenge to human health [2].

In the past several decades, the increased incidence of lifestyle-related diseases, such as diabetes mellitus (DM), cardiovascular disease (CVD), and hyperlipidemia, has made postoperative health care more challenging and complicated. DM is reported to be associated with the risk of postoperative complications and adverse cancer outcomes; a systematic review and several reports have suggested that DM or hyperglycemia is associated with an adverse overall survival (OS) and the disease-free survival in patients with solid tumors [3–6]. Moreover, postoperative complications following gastrectomy have recently been determined to be one of the factors associated with a poor prognosis of patients with GC because of either the postoperative systemic inflammatory response or host immunosuppression [7–9]. On the other hand, gastrectomy could improve type 2 DM in patients [10, 11]. Therefore, the blood glucose levels may have different implications in patients with GC who undergo gastrectomy.

Hemoglobin A1c (HbA1c) can reflect the glucose metabolism over the past ~ 3 months; therefore, it is widely accepted as gold-standard diagnostic marker for DM [12]. Although DM or poor postoperative glycemic control is reported to be a risk factor for short-term postoperative complications in patients with cancer [5, 13], the long-term prognostic impact of hyperglycemia or hypoglycemia remains debatable. Furthermore, the long-term prognostic impact of the HbA1c levels has not yet been evaluated in patients with GC.

This study investigated the clinical significance of the HbA1c levels in patients with GC by examining the relationship between the preoperative and postoperative HbA1c levels and the clinical outcomes according to the pathological stage.

## Methods

### Patients

Between January 2008 and May 2016, patients with stage II, III, or IV GC underwent gastrectomy at the Kyoto Prefectural University of Medicine Hospital, Japan. Patients whose preoperative HbA1c levels were not measured were excluded. For the remaining patients whose preoperative HbA1c levels were measured at approximately 4 weeks before gastrectomy, all measured HbA1c levels were converted to National Glycohemoglobin Standardization Program levels using the conversion formula [14].

The patients were divided into two groups, namely high preoperative HbA1c (HbA1c > 6.0%) and low preoperative HbA1c (HbA1c ≤ 6.0%) groups. The cutoff value was 6.0%, defined according to previous studies [10, 15–17]. Between the two groups, we investigated the correlation between the preoperative HbA1c levels and postoperative clinical outcomes including the prognosis according to pathological stage. Furthermore, we analyzed the preoperative and postoperative dynamics in HbA1c levels after gastrectomy. Postoperative complications were evaluated based on the Clavien–Dindo classification [18], and Clavien–Dindo classifications III, IV, and V were defined as “severe postoperative complications”. Pathological staging was performed using the 8th Union for International Cancer Control tumor, nodes, and metastases staging [19].

Postoperatively, for the first 2 years, all patients were followed up every 3–6 months, and the follow-up was continued for at least 5 years. Patients who were lost during follow-up were censored at the date of last contact/follow-up. Follow-up evaluation included physical examination, blood investigation, computed tomography (CT), and gastroscopy. Recurrence was confirmed by imaging, typically with CT, and histologically, if possible, via surgical biopsy, needle biopsy, or appropriate fluid cytology.

All procedures were performed in accordance with the Declaration of Helsinki of 1964 and later versions, and the ethical standards of the responsible committees on human experimentation, both institutional (approval no. ERB-C-1327) and national. Informed consent or a substitute was obtained from all patients prior to inclusion in the study.

### Statistical analysis

The statistical analysis was performed using s JMP software program version 10 (SAS Institute, Cary, NC, USA). Fisher’s exact probability test and the Chi-square test were used to compare categorical variables between the two groups, whereas nonparametric tests (Mann–Whitney *U*, Wilcoxon signed rank, and Kruskal–Wallis) were used for subgroup comparisons. OS and cancer-specific survival (CSS) were calculated using the Kaplan–Meier analysis, with the gastrectomy date as the starting point, and differences in survival were measured using the log-rank test. Univariate and multivariate analyses were performed using the Cox proportional hazards model. All statistical tests except for the paired tests were two-sided. *p* < 0.05 was considered to be statistically significant.

## Results

Overall, 306 patients with stage II, III, or IV GC underwent gastrectomy at our university hospital. Of these, 12 patients whose preoperative HbA1c levels were not measured were excluded. Finally, 294 patients with primary GC who underwent gastrectomy with D1 plus or D2 lymph node dissection were enrolled. Curative gastrectomy (R0) was performed for 245 patients with stage II and III GC, whereas palliative gastrectomy was performed for 49 patients with stage IV GC.

### Clinicopathological characteristics

Table 1 shows the association between the patients’ clinicopathological characteristics and their preoperative HbA1c levels. We found no significant difference in age, sex, and pathological backgrounds between the high and low preoperative HbA1c groups; however, the high preoperative HbA1c group had a higher body mass index (*p* < 0.0001) and required a longer operation time (*p* = 0.0281) than the low preoperative HbA1c group. Of the 294 patients, 35 (12%) developed severe postoperative complications that required surgical, endoscopic, or radiological intervention. The rate of severe postoperative complications in the high preoperative HbA1c group was significantly higher than that in the low preoperative HbA1c group (*p* = 0.0455).

**Table 1:** Association between the clinicopathological characteristics and the preoperative HbA1c levels (*n* = 294)

Variable	Preoperative HbA1c > 6.0%	Preoperative HbA1c ≤ 6.0%	*p* value
Total	73	221	
Age (years), median (range)	70 (48–85)	67 (29–94)	0.1022
Sex			0.1067
Male	55	144	
Female	18	77	
Body mass index (kg/m^2^), median (range)	23.5 (16.3–30.9)	21.3 (15.6–31.6)	** < 0.0001**
Neoadjuvant chemotherapy			0.8768
Present	5	14	
Absent	68	207	
Preoperative therapy for diabetes mellitus			** < 0.0001**
Oral	26	3	
Insulin	4	0	
Oral and insulin	3	0	
Absent	40	218	
Operation time (min), median (range)	270 (60–583)	236 (49–626)	**0.0281**
Blood loss during surgery (mL), median (range)	250 (5–2482)	226 (0–2290)	0.1560
Complication			**0.0455**
No complication	44	163	
Clavien–Dindo classification I/II	15	37	
Clavien–Dindo classification III/IV/V	14	21	
Tumor depth^a, b^			0.3047
T 1/2	15	34	
T 3/4	58	187	
Lymph node metastasis^a, b^			0.6552
Negative	17	46	
Positive	56	175	
Stage^a, b^			0.9975
II	30	90	
III	31	94	
IV	12	37	

Significant values are given in boldface

*HbA1c* hemoglobin A1c

^a^According to the 8th edition of the International Union Against Cancer tumor, node, metastasis (TNM) grading

^b^Pathological stage

### Postoperative clinical outcomes

**Fig. 1:**
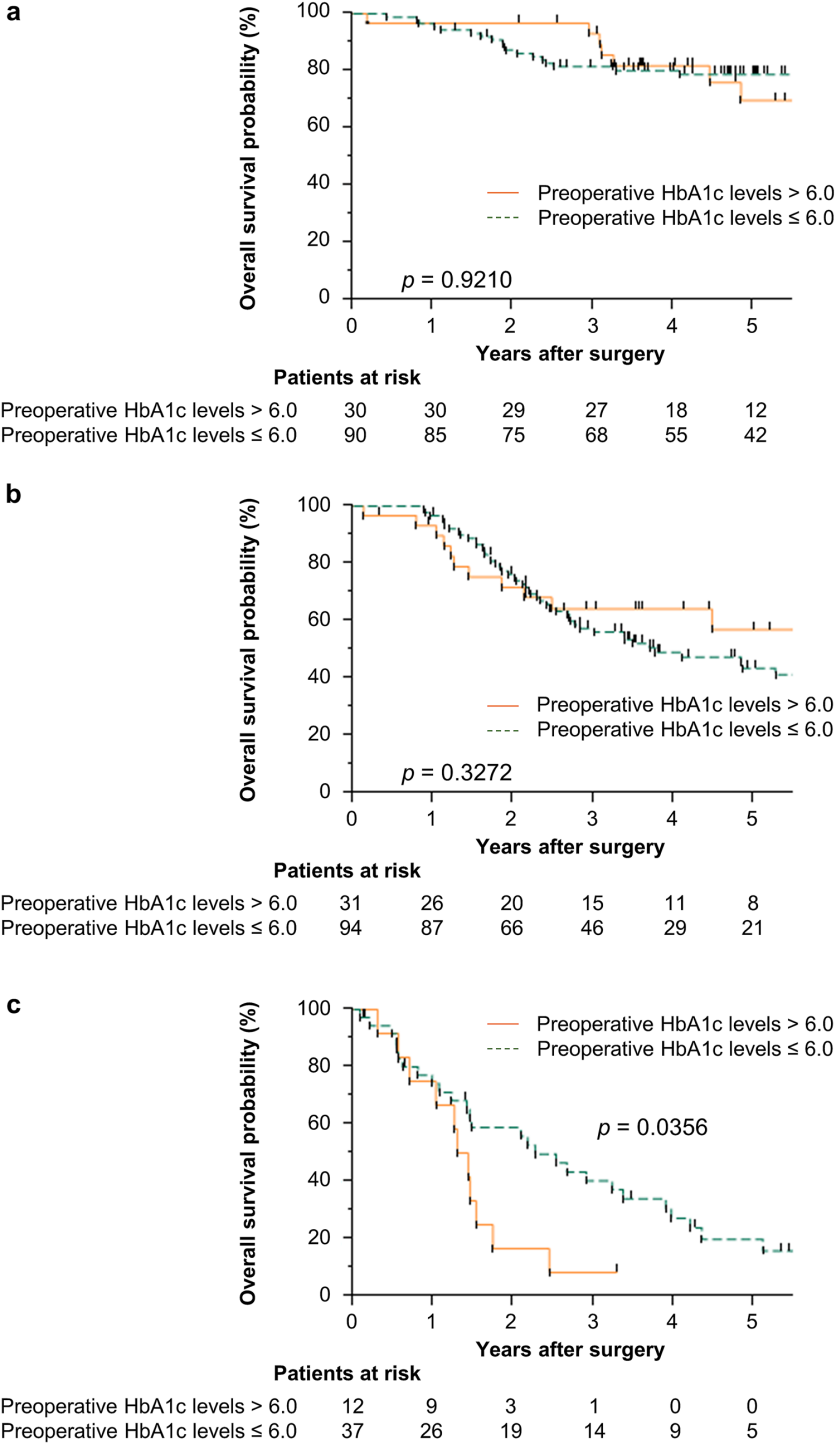
Overall survival in patients with gastric cancer Kaplan–Meier curves for overall survival rates of patients with gastric cancer according to preoperative hemoglobin A1c levels in stage II (**a**), stage III (**b**), and stage IV (**c**) gastric cancer

Figure 1 shows the prognostic impact of the preoperative HbA1c levels in patients with GC according to the pathological stage. The high preoperative HbA1c group had a significantly poor prognosis in patients with stage IV GC, although we found no significant difference between the two groups in patients with stage II or III GC (Fig. 1).

**Fig. 2:**
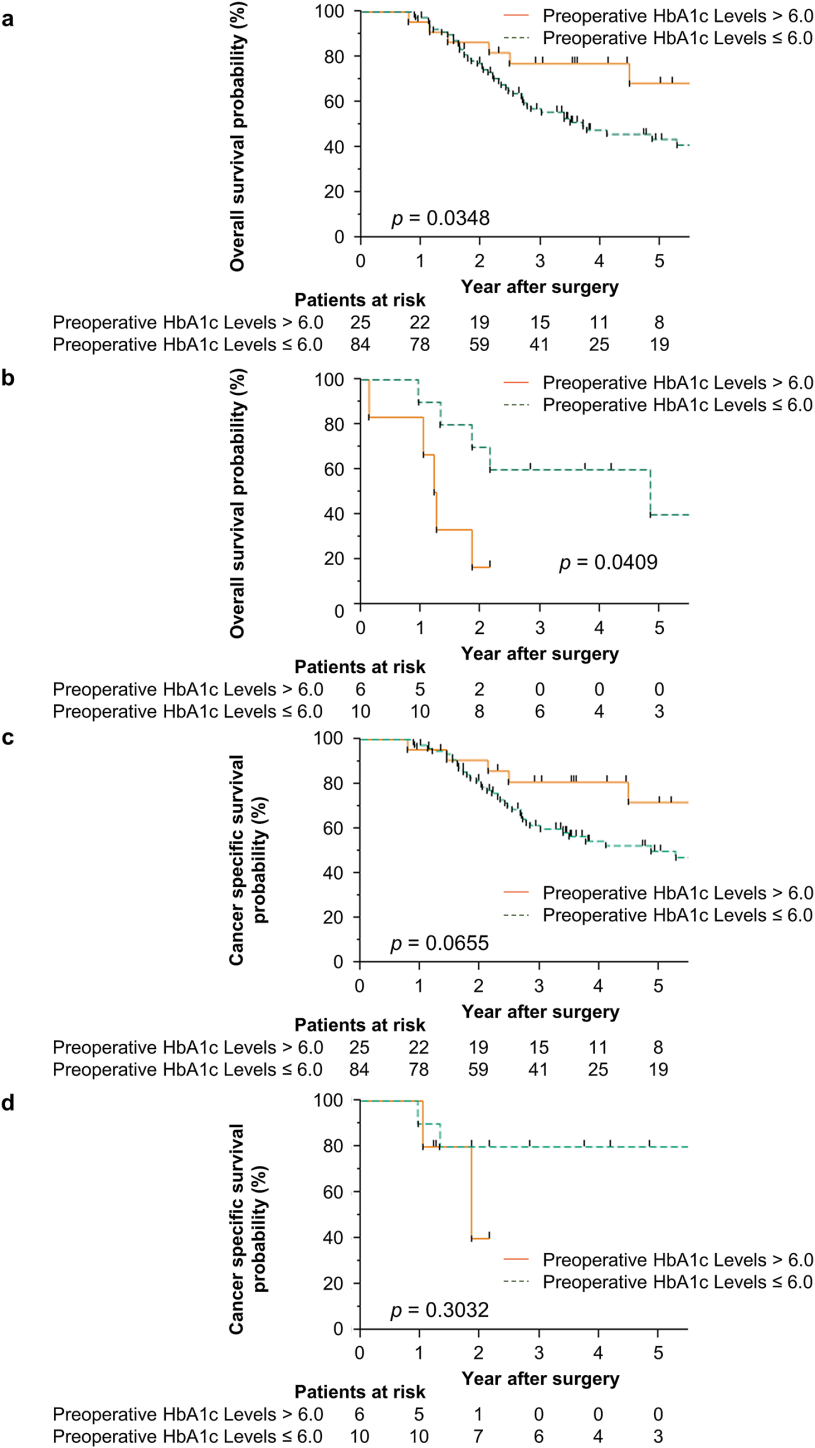
Overall survival and cancer specific survival in patients with stage III gastric cancer. Overall survival rates of patients with stage III gastric cancer according to preoperative hemoglobin A1c levels without (**a**) and with (**b**) severe postoperative complications. Cancer specific survival rates of patients with stage III gastric cancer according to preoperative hemoglobin A1c levels without (**c**) and with (**d**) severe postoperative complications

The subgroup analysis of the OS rates of patients with stage III GC according to the presence or absence of severe postoperative complications (Fig. 2a, b) showed that in patients with stage III GC with severe postoperative complications, the high preoperative HbA1c group had a significantly worse prognosis than the low preoperative HbA1c group (*p* = 0.0409). In patients without severe postoperative complications, the high preoperative HbA1c group had a significantly better prognosis than the low preoperative HbA1c group (*p* = 0.0348). The analysis of the CSS rates of patients with stage III GC according to the presence or absence of severe postoperative complications, although not significant, showed similar results and tendency (Fig. 2c, d). In patients with stage III GC without severe postoperative complications, the high preoperative HbA1c group tended to have a better prognosis than the low preoperative HbA1c group, whereas in patients with severe postoperative complications, the high preoperative HbA1c group tended to have a worse prognosis than the low preoperative HbA1c group (*p* = 0.0655 and 0.3032, respectively).

Table 2 shows the results of the Cox proportional hazard model in patients with stage III GC without severe postoperative complications. High preoperative HbA1c levels were significantly associated with a better prognosis in patients with stage III GC (hazard ratio [HR] = 0.43; 95% confidence interval [CI] = 0.16–0.94; *p* = 0.0319).

**Table 2:** The Cox proportional hazard regression analysis for overall survival in patients with stage III gastric cancer without severe postoperative complications

Variable	*n*	Univariate	Multivariate		
		*p* value	*p* value	HR	(95% CI)
Total	109				
Age (years)		0.3168			
< 70	58				
≥ 70	51				
Body mass index (kg/m^2^)		0.3588			
< 25.0	91				
≥ 25.0	18				
Neoadjuvant chemotherapy		0.6721			
Present	6				
Absent	103				
Operation time (min)		0.5716			
< 240	53				
≥ 240	56				
Blood loss (mL)		0.4549			
< 250	59				
≥ 250	50				
Preoperative HbA1c level (%)		**0.0348**	**0.0319**	0.43	(0.16–0.94)
≤ 6.0	84				
> 6.0	25				
Postoperative chemotherapy		0.6713			
Present	83				
Absent	26				
Stage^a,b^		** < 0.0001**	** < 0.0001**	3.75	(2.03–6.70)
IIIA/IIIB	91				
IIIC	18				

Significant values are given in boldface

*HR* hazard ratio, *CI* confidence interval, *HbA1c* hemoglobin A1c

^a^According to the 8th edition of the International Union Against Cancer tumor, node, metastasis (TNM) grading

^b^Pathological stage

### Without any preoperative therapy for diabetes mellitus

We investigated OS rates of 109 patients with stage III GC without any preoperative therapy for DM. Similar to the entire cohort, without severe postoperative complications, the high preoperative HbA1c group had a significantly better prognosis than the low preoperative HbA1c group, whereas in patients with severe postoperative complications, the high preoperative HbA1c group tended to have a worse prognosis than the low preoperative HbA1c group (*p* = 0.0458 and 0.0857).

### Preoperative and postoperative dynamics of HbA1c levels

**Fig. 3:**
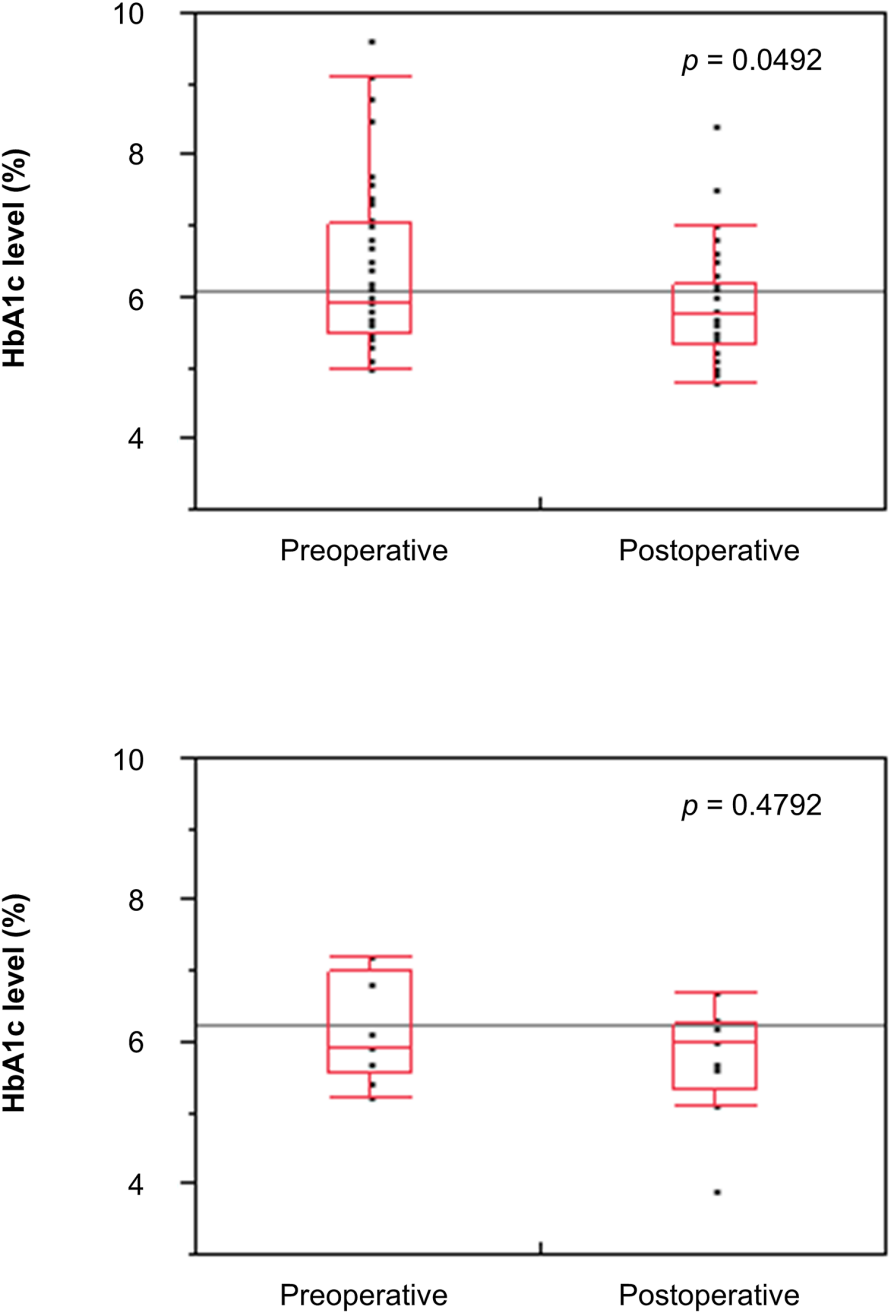
Comparison of preoperative and postoperative hemoglobin A1c levels. Comparison of preoperative and postoperative hemoglobin A1c levels in 46 patients with stage III gastric cancer without severe postoperative complications (top) and 9 patients with severe postoperative complications

A comparison of the preoperative and postoperative HbA1c levels in patients with stage III GC with and without severe postoperative complications (Fig. 3) showed a significant decrease in the HbA1c levels postoperatively in 46 patients without severe postoperative complications, but not in 9 patients with severe postoperative complications (*p* = 0.0492 and 0.4792, respectively). Further, 55.0% of patients with stage III GC without severe postoperative complications in the high preoperative HbA1c group had improved to normal HbA1c levels postoperatively. In addition, 41.9% of patients with stage III GC in the low preoperative HbA1c group had shown a decrease in the postoperative HbA1c levels (HbA1c < 5.4%).

## Discussion

The present study revealed a paradoxical correlation between the preoperative HbA1c levels and long-term outcomes in patients with GC who underwent gastrectomy. In patients with stage III GC with severe postoperative complications, the high preoperative HbA1c group had a worse prognosis than the low preoperative HbA1c group, whereas in patients with stage III GC without severe postoperative complications, the high preoperative HbA1c group had a significantly better prognosis than the low preoperative HbA1c group. In addition, we observed a similar tendency for patients without any preoperative therapy for DM. Similar results were not observed in patients with stage II GC, because these patients had a favorable prognosis regardless of the preoperative HbA1c levels. In patients with stage IV GC, the high preoperative HbA1c group had a significantly poor prognosis. The results suggested that hyperglycemic patients with residual cancer, such as stage IV GC, have a worse prognosis or that conversely advanced cancers affect their glucose metabolism by increasing insulin resistance [20]. Although it was difficult to clarify the process of the paradoxical correlation between preoperative HbA1c levels and long-term outcomes in patients with stage III GC, we believe that hyperglycemia, inflammation, and gastrectomy might have been associated with the process.

It is widely known that high HbA1c levels and hyperglycemia are related to adverse outcomes in surgical patients [21]. High HbA1c levels tend to be poor postoperative blood glucose control, prolonging postoperative hyperglycemia and leading to a high complication rate [17]. Postoperative hyperglycemia is associated with postoperative morbidity and mortality [22, 23] even in nondiabetic patients [24]. In our entire cohort, patients with high preoperative HbA1c levels had a significantly higher postoperative complication rate than those with low preoperative HbA1c levels. In general, hyperglycemia leads to body weight increase, adiposity, and general malaise caused by increased blood insulin levels. Obesity is also associated with hyperglycemia, endocrine signals, and chronic inflammation. Therefore, patients with hyperglycemia get caught up in this vicious cycle that creates favorable microenvironments in cancer cells, leading to further abdominal inflammation and malignant cell growth [25]. Our result that the CSS rates in patients with stage III GC were similar to the OS rates might imply that postoperative hyperglycemia and prolonged inflammation have a negative impact on cancer cells. Because glucose uptake and glycolysis are accelerated in cancer cells, hyperglycemia might affect their intracellular metabolism and lead to the production of inflammatory cytokines, and the systemic inflammatory responses caused might facilitate the growth and invasion of cancer cells [26–28]. Furthermore, postoperative inflammation triggers the development and sustenance of hyperglycemia by inducing insulin resistance and accelerating gluconeogenesis [29]. This description might be supported by the result of preoperative and postoperative dynamics of HbA1c levels in patients with stage III GC with and without severe postoperative complications in the present study, wherein HbA1c levels in patients with complication tended not to decrease postoperatively. Thus, high HbA1c levels or postoperative hyperglycemia might lead to postoperative complications, intra-abdominal inflammation, and eventually poor long-term outcomes.

However, with regard to gastrectomy, preoperative hyperglycemia or high HbA1c levels do not necessarily lead to a poor prognosis in patients with GC, because postoperative glucose levels decline on average after gastrectomy. There are several studies focusing on the glucose levels before and after bariatric surgery or gastrectomy, which resulted in a remarkable improvement in obesity and DM [11, 30, 31]. Schauer et.al [11] reported that patients who underwent gastrectomy had favorable glycemic control while using fewer diabetes medications and had a significant decrease in HbA1c levels. Thus, high preoperative HbA1c group might maintain appropriate postoperative glucose levels following gastrectomy if there are no complications, and the improvement in obesity and glycemic control might lead to suppression of chronic inflammation and favorable long-term outcomes. In patients with stage III GC without severe postoperative complications, the high preoperative HbA1c group had a significantly favorable prognosis compared with the low preoperative HbA1c group; however, preoperative therapy for DM is necessary because DM is associated with an increased risk of postoperative complications and adverse cancer outcomes [3–6]. In contrast, lower HbA1c levels are known to have a negative effect on patients. Goto et al. [32] showed that low the HbA1c levels are also associated with a higher risk of CVD. Although there was no significant difference in this study, a previous study reported that low pretreatment HbA1c levels might be associated with a poor prognosis for patients with esophageal cancer [33]. Therefore, the HbA1c levels are required to be within the normal range.

Postoperative complications lead to a poor prognosis of patients with GC [7, 34, 35]. Age, DM, liver cirrhosis, invasion of neighboring organs, combined resection, intraoperative transfusion, malnutrition, Billroth II reconstruction, surgical experience, and operation time were reported as risk factors of postoperative complications after gastrectomy [36]. Poor glycemic control is a widely known risk factor of postoperative complications [37]. In the present study, the postoperative complication rate of patients with low preoperative HbA1c levels was lower than that of patients with high preoperative HbA1c levels. However, a subgroup analysis in the group without postoperative complications showed that the low preoperative HbA1c group had a worse prognosis than the high preoperative HbA1c group, which might imply that the postoperative blood glucose levels decrease further thus leading to hypoglycemia in the low preoperative HbA1c group. Therefore, the management of postoperative hypoglycemia is also required for the patients with low preoperative HbA1c levels. Moreover, we have to make unwavering efforts during surgery to avoid postoperative complications by developing surgical techniques and devices (e.g., laparoscopy, robot, and surgical instruments), nutrition therapy, and optimal perioperative management especially for patients with high preoperative HbA1c levels.

The present study is associated with some limitations. First, it was a retrospective single-center study, and the study cohort was relatively small sample size. Second, we evaluated only the preoperative HbA1c levels, without considering the fasting blood glucose or perioperative serum glucose levels. We could not investigate the postoperative HbA1c levels in patients with low preoperative HbA1c levels because of the unavailability of data details. In addition, there was some bias in this cohort with regard to the HbA1c levels of patients that were within a relatively acceptable range. Thus, the preliminary findings of the present study should be validated in a larger patient cohort and accumulation of cases with systematical management would be required to address these limitations.

In summary, the prognosis of patients with high preoperative HbA1c levels greatly depends on the presence or absence of postoperative complications. To avoid postoperative complications, optimal perioperative management and personalized treatment would be critical, particularly for patients with stage III GC having high preoperative HbA1c levels.
